# Supplementary material for: Efficacy and safety of Saireito (TJ-114) in patients with atrial fibrillation undergoing catheter ablation procedures: A randomized pilot study
Source: PLoS One. 2024 Aug 1;19(8):e0307854. doi: 10.1371/journal.pone.0307854 (PMC11293677; doi:10.1371/journal.pone.0307854)
Supplement: S1 Table — (DOCX) [file pone.0307854.s001.docx]

**Table S1: Clinical outcomes in the subgroups**

| **Variables** | | **Frequent atrial arrhythmias**  **N of patients with events/N of patients** | | | **Adverse symptoms**  **N of patients with events/N of patients** | | |
| --- | --- | --- | --- | --- | --- | --- | --- |
|  |  | **Saireito** | **Control** | **P-value** | **Saireito** | **Control** | **P-value** |
| **Overall** |  | 3.1±4.6 (N=50) | 5.2±9.4 (N=50) | 0.17 | 9/50 (18.0%) | 1/50 (2.0%) | 0.008 |
| **Age** | ≥ 70yr | 3.6±5.3 (N=29) | 7.0±11.4 (N=31) | 0.15 | 6/29 (20.7%) | 0/31 (0.0%) | 0.01 |
|  | < 70yr | 2.4±3.6 (N=21) | 2.3±2.9 (N=19) | 0.84 | 3/21 (14.3%) | 1/19 (5.3%) | 0.34 |
| **Gender** | Female | 3.5±5.8 (N=25) | 5.7±13.2 (N=22) | 0.45 | 4/25 (16.0%) | 1/22 (4.6%) | 0.22 |
|  | Male | 2.8±3.0 (N=25) | 4.8±4.9 (N=28) | 0.08 | 5/25 (20.0%) | 0/28 (0.0%) | 0.02 |
| **Body weigh** | ≥60 kg | 3.2±3.6 (N=24) | 6.2±7.4 (N=24) | 0.08 | 4/24 (16.7%) | 0/24 (0.0%) | 0.05 |
|  | <60 kg | 3.1±5.5 (N=26) | 4.3±11.0 (N=26) | 0.61 | 5/26 (19.2%) | 1/26 (3.9%) | 0.10 |
| **AF type** | Paroxysmal AF | 2.9±4.9 (N=41) | 4.0±6.0 (N=41) | 0.34 | 7/41 (17.1%) | 1/41 (2.4%) | 0.03 |
|  | Non-paroxysmal AF | 4.4±3.2 (N=9) | 10.6±17.9 (N=9) | 0.33 | 2/9 (22.2%) | 0/9 (0.0%) | 0.24 |
| **AF interval** | ≥3 year | 4.9±7.5 (N=14) | 7.1±14.8 (N=14) | 0.63 | 1/14 (7.1%) | 0/14 (0.0%) | 0.50 |
|  | <3 years | 2.4±2.7 (N=36) | 4.5±6.4 (N=36) | 0.09 | 8/36 (22.2%) | 1/36 (2.8%) | 0.01 |
| **EHRA score** | ≥3 | 2.4±4.8 (N=34) | 5.8±10.8 (N=36) | 0.10 | 6/34 (17.7%) | 1/36 (2.8%) | 0.04 |
|  | <3 | 4.8±3.8 (N=16) | 3.8±4.5 (N=14) | 0.50 | 3/16 (18.8%) | 0/14 (0.0%) | 0.14 |
| **Previous heart failure** | Yes | 1.0±0.0 (N=3) | 28.0±39.6 (N=2) | 0.29 | 0/3 (0.0%) | 0/2 (0.0%) | - |
|  | No | 3.3±4.7 (N=47) | 4.3±6.0 (N=48) | 0.38 | 9/47 (19.2%) | 1/48 (2.1%) | 0.007 |
| **Chronic kidney disease** | Yes | 3.0±2.5 (N=25) | 7.5±12.7 (N=24) | 0.09 | 2/25 (8.0%) | 0/24 (0.0%) | 0.26 |
|  | No | 3.3±6.1 (N=25) | 3.1±4.1 (N=26) | 0.91 | 7/25 (28.0%) | 1/26 (3.9%) | 0.02 |
| **BNP** | ≥120 pg/ml | 3.5±2.7 (N=16) | 6.6±8.6 (N=14) | 0.19 | 1/16 (6.3%) | 0/14 (0.0%) | 0.53 |
|  | <120 pg/ml | 3.0±5.4 (N=33) | 3.4±4.5 (N=30) | 0.75 | 8/33 (24.2%) | 1/30 (3.3%) | 0.02 |
| **NT-pro BNP** | ≥600 pg/ml | 3.8±2.6 (N=12) | 10.1±15.8 (N=14) | 0.19 | 0/12 (0.0%) | 0/14 (0.0%) | - |
|  | <600 pg/ml | 2.4±3.3 (N=34) | 3.4±4.3 (N=34) | 0.24 | 9/34 (26.5%) | 1/34 (2.9%) | 0.007 |
| **Troponin I** | ≥5.0 pg/ml | 2.7±2.9 (N=10) | 8.5±14.7 (N=17) | 0.23 | 1/10 (10.0%) | 0/17 (0.0%) | 0.37 |
|  | <5.0 pg/ml | 2.3±5.0 (N=40) | 3.6±4.4 (N=32) | 0.76 | 8/40 (20.0%) | 1/32 (3.1%) | 0.03 |
| **C-reacting protein** | ≥1.0 mg/dl | 4.3±6.1 (N=19) | 5.6±14.6 (N=14) | 0.73 | 3/19 (15.8%) | 0/14 (0.0%) | 0.18 |
|  | <1.0 mg/dl | 2.5±3.4 (N=31) | 5.1±6.7 (N=36) | 0.05 | 6/31 (19.4%) | 1/36 (2.8%) | 0.03 |
| **Cryoballoon** | Yes | 3.1±5.3 (N=34) | 4.3±6.6 (N=32) | 0.44 | 7/34 (20.6%) | 1/32 (3.1%) | 0.03 |
|  | No | 3.1±3.0 (N=16) | 6.8±13.1 (N=18) | 0.28 | 2/16 (12.5%) | 0/18 (0.0%) | 0.22 |

AF=atrial fibrillation; BNP=brain natriuretic peptides; NT-proBNP=N-terminal pro-brain natriuretic peptides
