# Supplementary figures and images for: Efficacy and safety of Saireito (TJ-114) in patients with atrial fibrillation undergoing catheter ablation procedures: A randomized pilot study
Source: PLoS One. 2024 Aug 1;19(8):e0307854. doi: 10.1371/journal.pone.0307854 (PMC11293677; doi:10.1371/journal.pone.0307854)

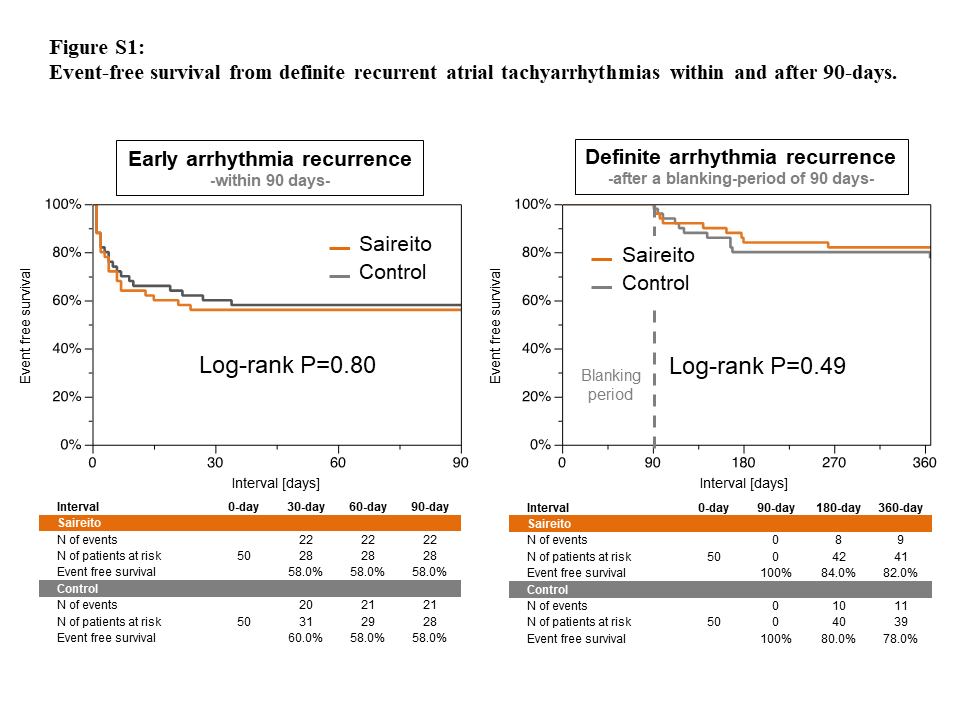

Supplement: S1 Fig — (TIF) [file pone.0307854.s002.tif]

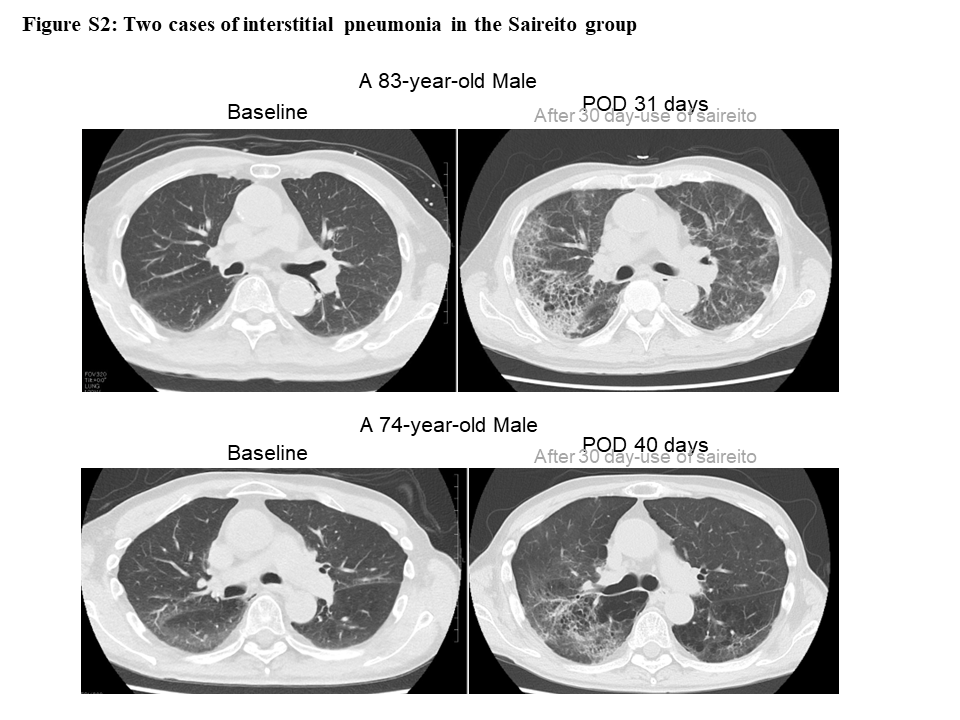

Supplement: S2 Fig — (TIF) [file pone.0307854.s003.tif]
